# Developing an in-vivo physiological porcine model of inducing acute atraumatic compartment syndrome towards a non-invasive diagnosis using shear wave elastography

**DOI:** 10.1038/s41598-021-01405-0

**Published:** 2021-11-08

**Authors:** Jong Woo Kang, Jong Woong Park, Tae Hyun Lim, Keun Tae Kim, Song Joo Lee

**Affiliations:** 1 Department of Orthopaedic Surgery, Korea University Ansan Hospital, Ansan, Republic of Korea; 2 Department of Orthopaedic Surgery, Korea University Anam Hospital, Seoul, Republic of Korea; 3 Center for Bionics, Biomedical Research Institute, Korea Institute of Science and Technology, Seoul, Republic of Korea; 4 Division of Bio-Medical Science and Technology, KIST School, Korea University of Science and Technology (UST), Seoul, Republic of Korea

**Keywords:** Health care, Medical research

## Abstract

Compartment syndrome (CS) is a pathological event caused by elevated intracompartmental pressure (ICP); however, changes from the onset of inducing atraumatic CS remained unclear. The study aimed to investigate the physiological changes in a newly developed in vivo porcine acute atraumatic CS model. CS was induced by ischemia–reperfusion injury in the left hind leg of fourteen pigs divided into an echogenicity group (EG) and a shear wave elastography group (SEG). Echogenicity was measured in EG, and shear elastic modulus (SEM) was measured in SEG seven times before, at the onset of inducing CS, and every 30 min after the onset over eight hours. Simultaneously, ICP, blood pressure, and muscle perfusion pressure (MPP) were also measured in both groups. Our results indicate that SEM of the experimental leg in SEG significantly increased as CS developed compared to the control leg (p = 0.027), but no statistical difference in the echogenicity in EG was found between the experimental leg and control leg. There were also significant correlations between SEM and ICP (p < 0.001) and ICP and MPP (p < 0.001). Our method and findings can be a basis to develop a non-invasive diagnostic tool using a shear wave elastography for atraumatic CS.

## Introduction

Compartment syndrome (CS) is defined as impaired muscle perfusion due to increased pressure in the closed muscle compartment^1^. Untreated CS can lead to irreversible muscle damage and disability. Prompt recognition of CS is the most significant procedure to prevent irreversible changes^2^. Nevertheless, CS is typically induced by a traumatic event^1,3^; however, it can also occur due to atraumatic causes like excessive physical training, medical history of anticoagulants, bleeding disorders, vascular obstruction, recovered hypovolemia, and prolonged cardiac or vascular surgery^2^. Especially, in acute atraumatic CS, ischemia–reperfusion injury of a limb is the occasional and representative cause of acute atraumatic CS in general hospitals^4–7^. This can occur anytime in hemodynamically unstable conditions such as shock, prolonged vascular surgery, heart surgery, and so on. Most patients suffering from these conditions should be managed in the intensive care unit (ICU) and may not complain about their symptoms due to poor general conditions or unconsciousness^2,8–11^. The favorable prognosis of CS can be achieved through accurate and timely diagnosis. Patients with CS suffer pallor (pale skin tone), pain, pulselessness (faint pulse), paresthesia (numbness feeling), and paralysis (weakness with movements) of the affected extremity (5P), and 5P traditionally has been used to diagnose CS^12–15^. The clinical diagnosis with only the 5Ps was inaccurate and confusing^12–15^. The current gold standard in diagnosing CS is to directly measure intracompartmental pressure (ICP) with invasive analog or digital devices^12–14^. Although invasive techniques are useful for measuring actual ICP, serious shortcomings exist^16–19^. They can induce iatrogenic injury and even elevate the ICP of the affected extremity^16,17,19^. Furthermore, repetitive measurement or continuous monitoring of ICP is more dangerous and impossible with these invasive techniques, although timely diagnosis is essential in cases of suspected CS to avoid delay in its diagnosis and treatment^16,20^.

The increased ICP can cause sequential physiologic alterations of muscle like decreased muscle perfusion, muscle ischemia, muscle necrosis, inflammatory muscle swelling, and subsequent increased ICP^5,12^. Ultrasound can detect various changes, such as swelling, stiffness, and ischemia, in muscles in CS non-invasively. The swollen muscle alters echogenicity on ultrasound and increases muscle stiffness^21^. The increase in muscle stiffness can increase muscle elasticity that can be measured using the ultrasound image machine^22^. The increased ICP decreased arterial blood flow that can be measured using Doppler measurements^5,18,23^. Although a recent study demonstrated that there is a significant correlation between the ICP and shear modulus obtained from shear wave elastography (SWE) among healthy individuals^24^, there are no comprehensive studies simultaneously investigating echogenicity, muscle elasticity, and arterial blood flow in an in vivo acute atraumatic CS model. There is only one study in CS that investigated the relationship between the ICP and muscle elasticity using SWE for the first time using a dead turkey limb model; however, the absolute muscle stiffness has not been investigated^25^. Understanding comprehensive physiological changes associated with CS can be further possible by measuring these values simultaneously since there can be a significant relationship between the muscle perfusion pressure associated with blood pressure and ICP. Thus, the authors devised a physiologic porcine leg model for acute atraumatic CS and intended to investigate the correlations of ICP and changes in muscle echogenicity, elasticity, and arterial blood flow velocity on ultrasound during acute CS. Our findings and methods in this study can be used to further develop a new non-invasive sonographic technique that is safe, accurate, and reproducible in measuring and monitoring ICP in the future.

## Results

**Figure 1:**
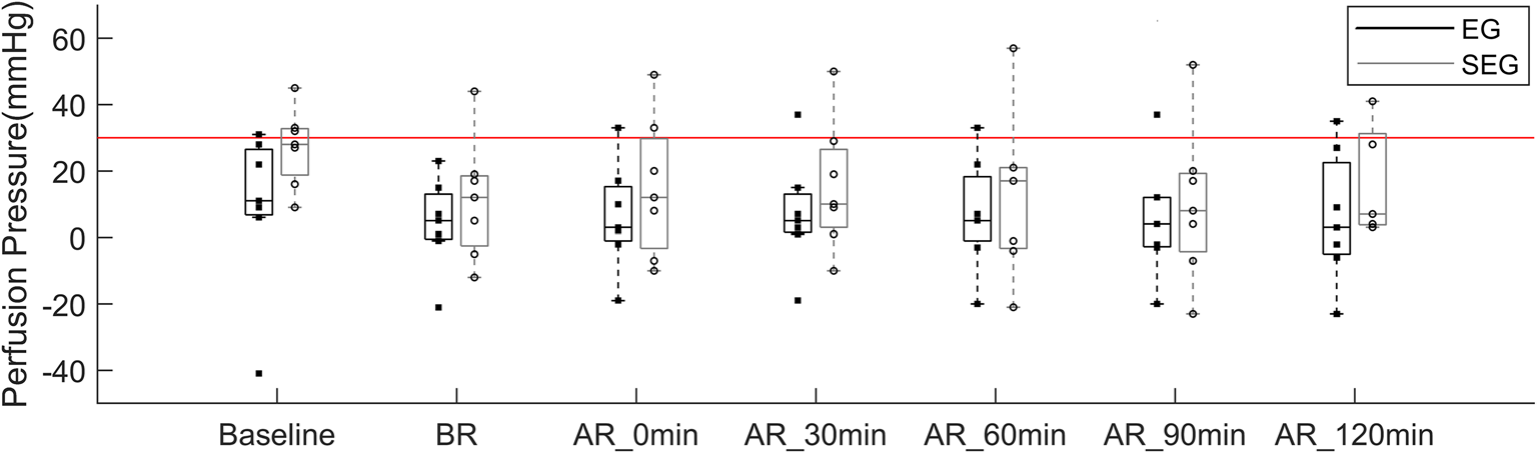
Perfusion pressure of the experimental leg between the EG (black bar plot) and SEG (gray bar plot) at each time point (Baseline, BR, AR_0 min, AR_30 min, AR_60 min, AR_90 min, and AR_120 min). Each dot indicates each data point in each group. Each bar plot indicates 25th (Q1), 50th (Q2), and 75th (Q3) percentiles of the data. The whisker indicates Q3 + 1.5 × (Q3–Q1), and Q1 − 1.5 × (Q3–Q1). The red line indicates the 30 mmHg showing the cut-off value for the induced CS (< 30 mmHg).

Wilcoxon rank-sum tests revealed that there were no significant differences in the ICP and perfusion pressure of the experimental legs between the Echogniecity Group (EG) and Shear wave elastography group (SEG) at each time point. As seen from Fig. 1, CS was induced for six out of seven pigs in EG and six out of seven pigs in SEG starting from AR_0min.

### Echogenicity group

**Figure 2:**
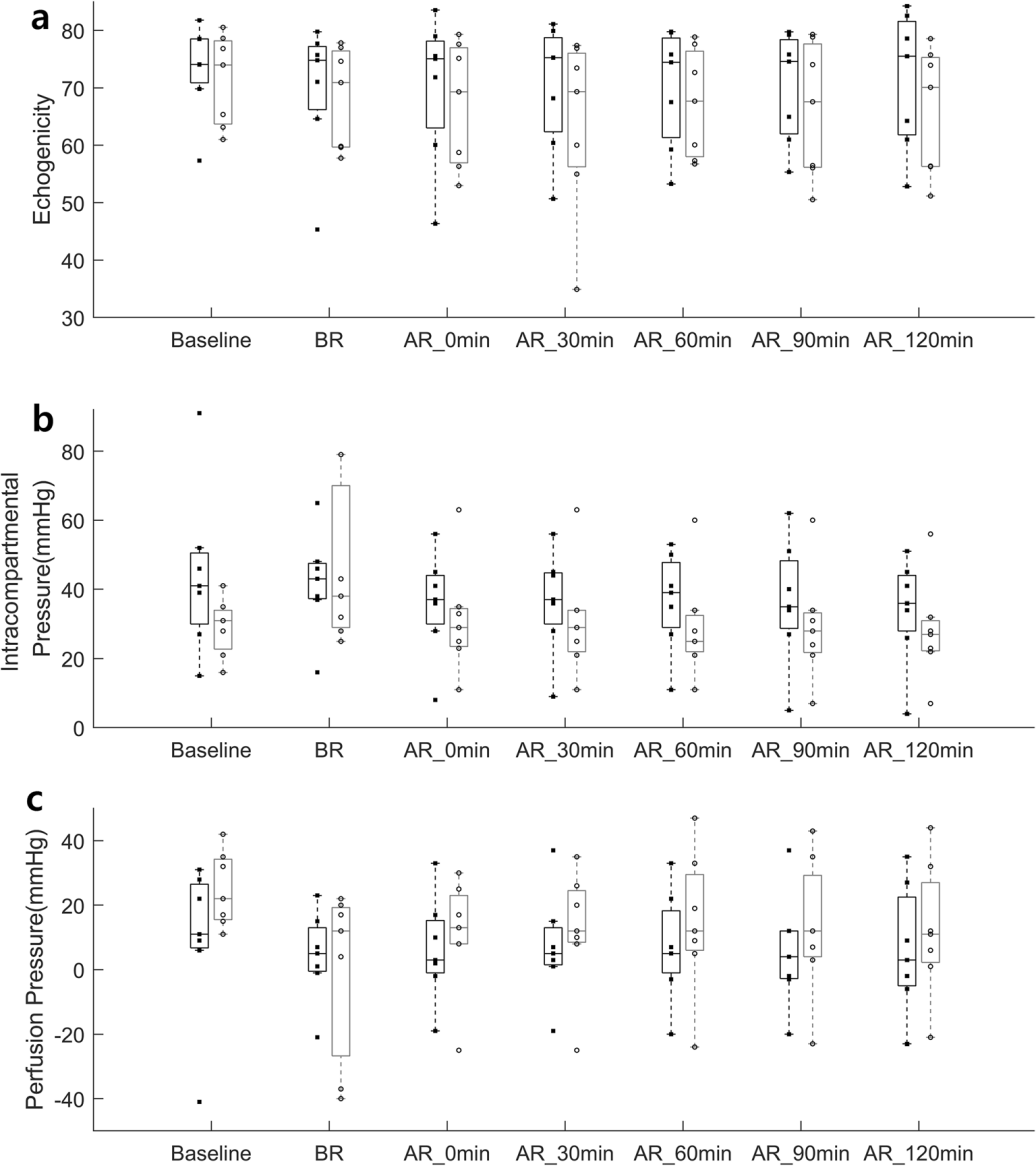
**(a)** Echogenicity, **(b)** intracompartmental pressure, and **(c)** perfusion pressure between the experimental leg (black bar plot) and the control leg (gray bar plot) at each time point (Baseline, BR, AR_0 min, AR_30 min, AR_60 min, AR_90 min, and AR_120 min). Each dot indicates each data point in each group. Each bar plot indicates 25th (Q1), 50th (Q2), and 75th (Q3) percentiles of the data. The whisker indicates Q3 + 1.5 × (Q3–Q1), and Q1 − 1.5 × (Q3–Q1).

**Figure 3:**
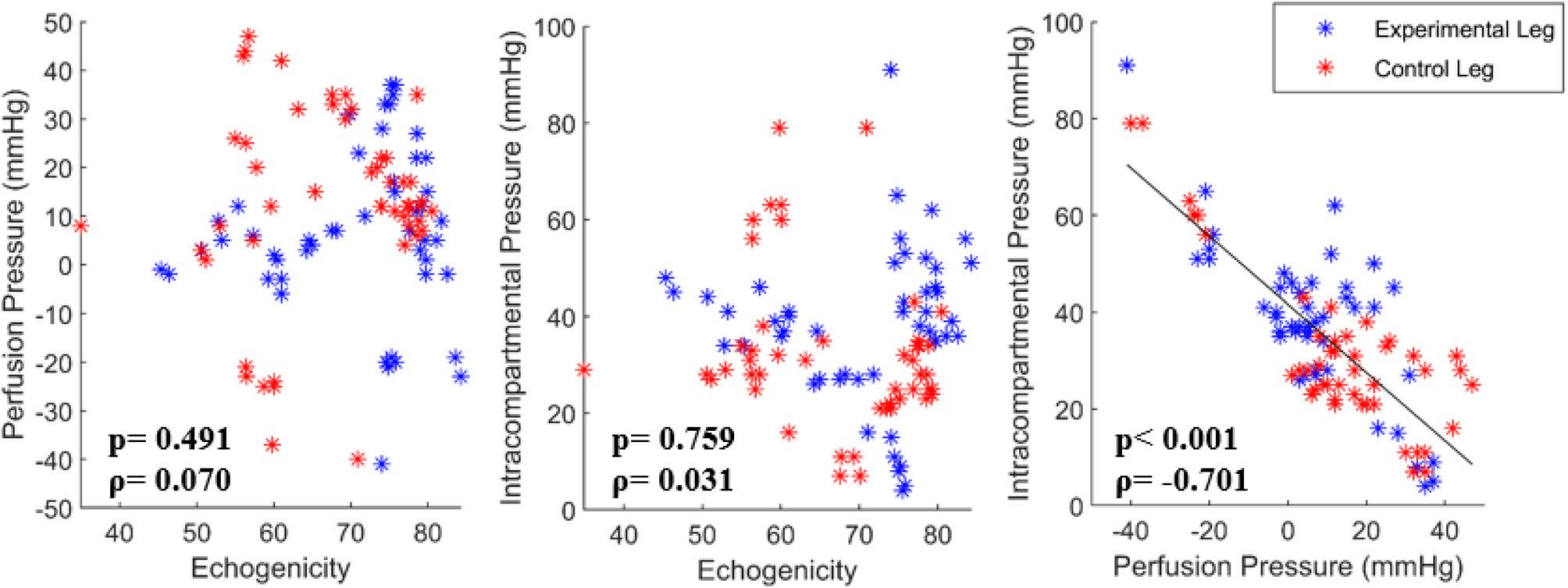
Echogenicity vs. intracompartmental pressure, echogenicity vs. perfusion pressure, and intracompartmental pressure vs. perfusion pressure in the echogenicity group (EG). Each blue asterisk indicates each data point from all time points of the experimental leg, and each red asterisk indicates each data point from all time points of the control leg in the EG.

Two-way repeated measure ANOVA analysis of the ranked data revealed that the maximum blood flow velocity showed a significant effect of condition (p=0.012), and interaction of time and condition (p < 0.001) and the minimum blood flow velocity showed a significant effect of condition (p = 0.001), and interaction of time and condition (p =0.019). As seen from Table 1, post hoc analysis of the ranked data revealed that the maximum blood flow velocity showed significant differences between the experimental leg and control leg at baseline (p = 0.035), AR_60min (p = 0.049), AR_90min (p=0.028), and AR_120min (p=0.014). As seen from Table 1, post hoc analysis of the ranked data revealed that the minimum blood flow velocity showed significant differences between the experimental leg and control leg at BR (p < 0.007), AR_0 min (p = 0.049), AR_90min (p=0.021), and AR_120min (p=0.007). As seen from Fig. 2, there were no statistical differences between the experimental leg and control leg on the echogenicity, perfusion pressure, and ICP at each time point. As seen from Fig. 3, Spearman's rank correlation analysis revealed that there was a significant correlation between the perfusion pressure and the ICP (p < 0.001, ρ = −0.701); however, there was no correlation between the echogenicity and the ICP, echogenicity, or perfusion pressure.

**Table 1:** Blood flow maximum and minimum velocity in the echogenicity group.

Measure	Experimental leg, mean (SD)	Control leg, mean (SD)
**Blood flow maximum velocity (cm/s)**		
Baseline	0.34 (0.91)	21.11 (13.09)*
BR	0.19 (0.49)	23.99 (12.11)
AR_0 min	7.14 (7.31)	23.56 (12.86)
AR_30 min	7.03 (5.92)	22.23 (12.62)
AR_60 min	7.26 (4.68)	20.43 (9.41)*
AR_90 min	6.09 (4.05)	18.56 (8.78)*
AR_120 min	6.81 (4.95)	18.04 (8.80)*
**Blood flow minimum velocity (cm/s)**		
Baseline	0.16 (0.42)	4.54 (2.90)
BR	0.20 (0.49)	4.69 (2.39)*
AR_0 min	1.93 (1.84)	5.36 (3.22)*
AR_30 min	(2.04)	5.06 (3.43)
AR_60 min	2.57 (0.98)	4.89 (3.31)
AR_90 min	2.01 (1.12)	4.34 (2.80)*
AR_120 min	1.90 (1.06)	4.43 (2.24)*

*SD* standard deviation, *BR* before reperfusion of the common iliac artery, *AR* after reperfusion of the common iliac artery.

*p < 0.05 between the experimental leg and control leg at the corresponding time point.

### Shear wave elastography group

**Figure 4:**
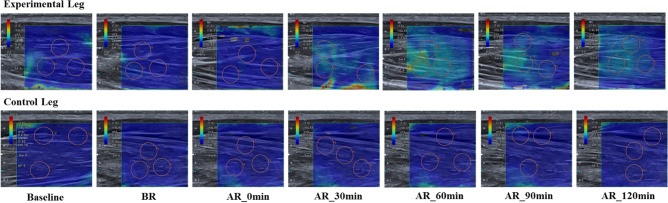
A representative shear wave elastography images of the experimental leg and control leg at each time point. Every three pink circles per image indicate the random region of interest for further data analysis.

Representative SWE images at each time point between the experimental leg and the control leg are illustrated in Fig. 4.

**Figure 5:**
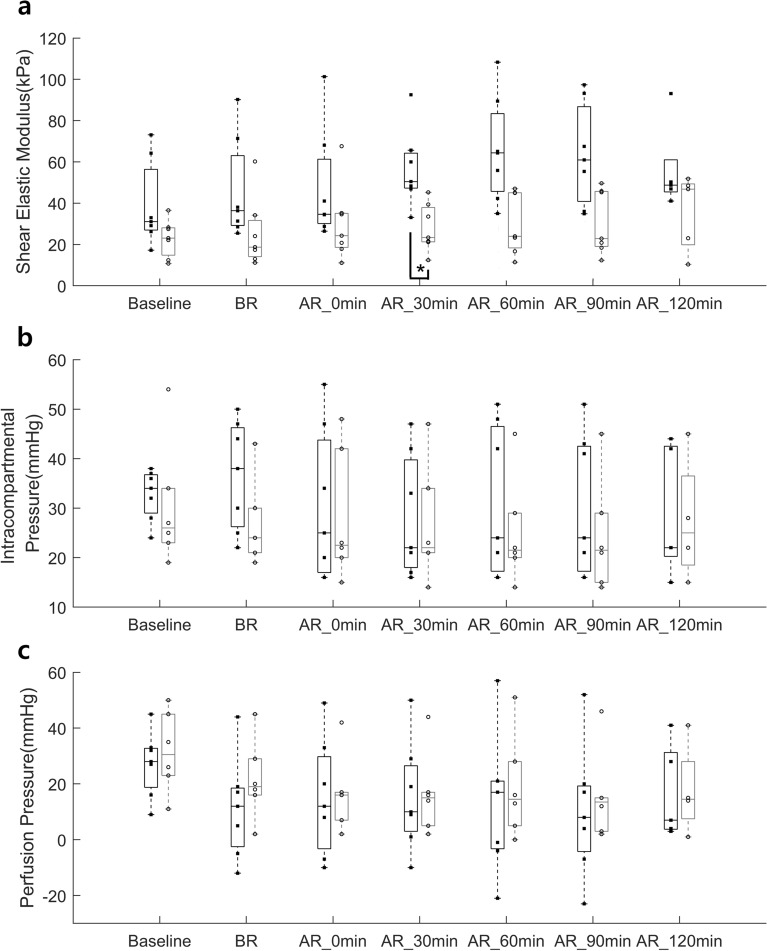
**(a)** Shear elastic modulus, **(b)** intracompartmental pressure, and **(c)** perfusion pressure between the experimental leg (black barplot) and the control leg (gray barplot) at each time point (baseline, BR, AR_0 min, AR_30 min, AR_60 min, AR_90 min, and AR_120 min). Each dot indicates each data point in each group. Each bar plot indicates 25th (Q1), 50th (Q2), and 75th (Q3) percentiles of the data. The whisker indicates Q3 + 1.5 × (Q3–Q1), and Q1 − 1.5 × (Q3–Q1).

**Figure 6:**
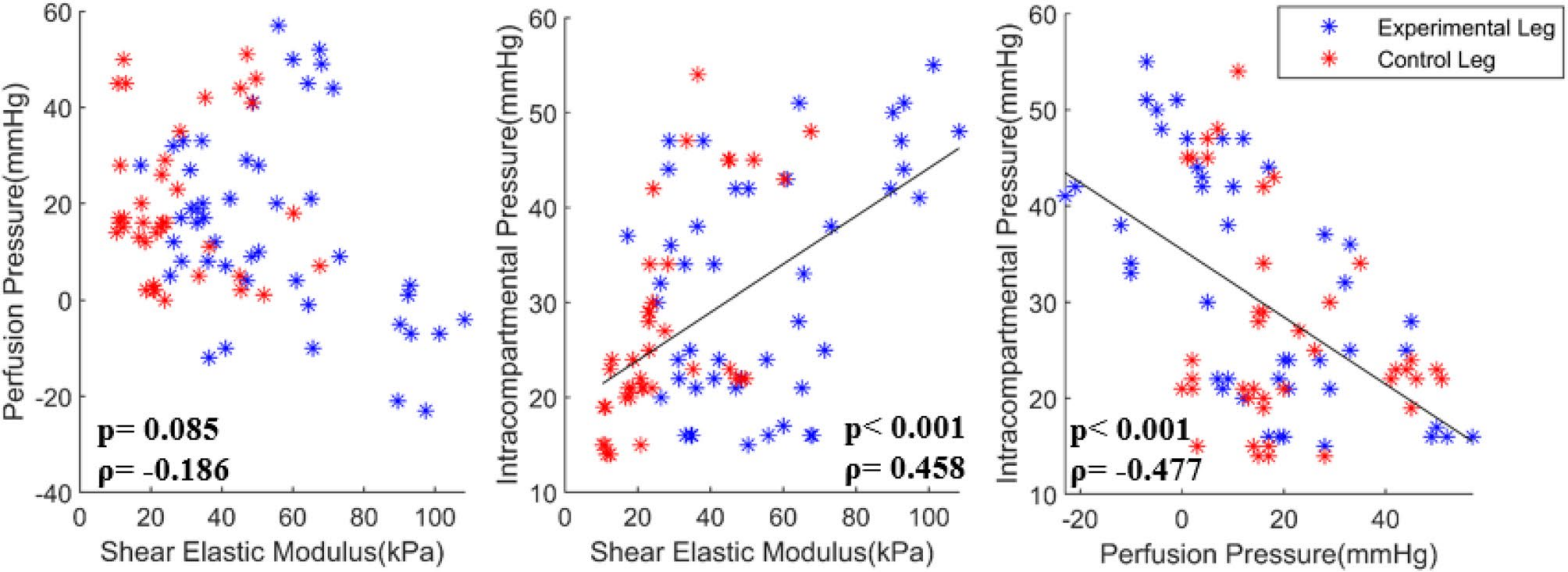
Shear elastic modulus versus intracompartmental pressure, shear elastic modulus versus perfusion pressure, and intracompartmental pressure versus perfusion pressure in the shear wave elastography group. Each blue asterisk indicates each data point from all time points of the experimental leg, and each red asterisk indicates each data point from all time points of the control leg in the shear wave elastography group.

Two-way repeated measure ANOVA analysis of the ranked data revealed that the shear elastic modulus showed a significant effect of condition (p=0.027) and time (p=0.003). As seen from Fig. 5, post hoc analysis of the ranked data revealed that the shear elastic modulus showed a significant difference between the experimental leg and control leg at AR_30 min (p = 0.028). However, there were no statistical differences between the experimental leg and control leg on the perfusion pressure and ICP. As seen from Fig. 6, Spearman's rank correlation analysis revealed that there was a significant correlation between the elastography and the ICP (p < 0.001, ρ = 0.458) and the perfusion pressure and the ICP (p < 0.001, ρ = − 0.477); however, no correlation between the elastography and perfusion pressure.

## Discussion

This is the first study to investigate comprehensive measurements related to detecting CS using an in vivo porcine acute atraumatic CS leg model. Our developed model is unique and novel since the induced CS is physiologically quite similar to an acute atraumatic CS of a human limb^26,27^. Our results indicate that shear elastic modulus significantly increases as CS develops compared to the control leg. There were also significant correlations between the shear elastic modulus and the ICP. Although the pattern of higher echogenicity was observed in the experimental leg compared to the control leg after reperfusion, it was not sensitive enough compared to the shear elastic modulus value to detect the CS.

In the present study, the CS was triggered by ischemia-reperfusion injury of the porcine leg muscle without any artificial manipulation into the target compartment, whereas in previous studies the CS was made by various controls, such as direct infusing fluid into a target compartment or deliberate mechanical damage of muscle^21,25,28–30^. Therefore, our animal model is favorable for measuring the ultrasonographic changes in pure muscle properties during CS. Thereby, our model shows a significant difference from others^21,25,28–30^. Reperfusion after tissue ischemia generates reactive oxygen species and activates neutrophils^31,32^. They cause muscle necrosis, swelling, and finally elevate ICP^31,32^. This model has pathophysiology similar to that of CS after revascularized limb. Based on our results in the EG, as demonstrated in Table 1, our animal model successfully blocked the arterial blood flow for intending to induce CS.

Clinically, the ICP above 30 mmHg or perfusion pressure less than 30 mmHg indicates the CS is induced in humans^33^. In our study, perfusion pressure was calculated by subtracting the ICP from the diastolic blood pressure. Some literature reported that perfusion pressure less than 30 mmHg is a useful clinical parameter for CS diagnosis^33^. Using these criteria, in our case, CS was induced for six out of seven pigs in the EG and six out of seven pigs in the SEG starting from AR_0min. No significant differences in the ICP and perfusion pressure of the experimental legs between the EG and SEG indicate that our model is valid. However, careful interpretation of our results is needed. Because ICP and perfusion pressure can also be influenced by the blood pressure of the pig throughout the long experiment, it is possible that although the ICP is not above 30 mmHg, earlier physiological changes associated with CS may be induced since the perfusion pressure after the reperfusion decreased in the experimental leg compared to the control leg. That was the reason that we also investigated measurements from the control leg and the experimental leg so that at each time point both conditions can be compared. Previous studies also reported that the variability in reported ICP thresholds inducing CS might be due to the differences in the perfusion state of the limbs^33^. Indeed, over seven time points, the diastolic blood pressure decreases from 53.9 ± 7.7 mmHg at the baseline to 40.0 ± 15.1 mmHg at the 120 min after reperfusion in the echogenicity group, and from 59.9 ± 10.3 mmHg at the baseline to 39.3 ± 15.4 mmHg at the 120 min after reperfusion in the shear wave elastography group. Thus, the variation of ICP might be associated with each pig’s blood pressure. Although the ICP values in our study are lower than the previous study^34^ reporting above 40 mmHg ICP right after ballon removal and gradual decrease until near the 40 mmHg ICP, the patterns of increase in the ICP in the experimental leg compared to the control leg were similar to our study. Considering that the diastolic blood pressure of the pigs became lower than at the beginning of the experiments, our results are valid and reasonable.

Previous literature suggests SWE as a diagnostic tool for various diseases such as liver, breast, prostate, thyroid, and musculoskeletal pathologies^35–39^. A recent study investigated the possibility of ultrasound SWE for measuring the ICP of CS in a turkey hind limb model^38^. In this study, increased shear wave speed in the lower limbs of six turkeys was observed as the ICP was increased. In the model, the ICP was artificially induced by infusing fluid through a balloon catheter in the dead turkey’s hind limb; thus, the CS was not naturally induced and the physiological changes in muscle during real CS were not considered. Furthermore, the absolute shear modulus of elasticity was not reported. Although it is difficult to make a direct comparison of the shear wave velocity from the shear elastic modulus, the latter increases with the increase in the shear wave velocity since shear waves propagate faster through stiffer tissue^39–41^. Thereby, our finding of significant differences in shear elastic modulus in the experimental leg compared to the control leg indicated that there were changes in muscle stiffness due to the ischemia-reperfusion injury in the porcine leg. Furthermore, a significantly positive correlation between the shear elastic modulus and the ICP indicated that the shear elastic modulus could be used non-invasively to further develop a diagnostic tool for CS.

While a statistically significant positive correlation was observed between the shear elastic modulus and the ICP, no correlation was detected between echogenicity and ICP. Although there was a trend of higher echogenicity in the experimental leg compared to the control leg as CS was induced, the results were not statistically significant, while a significant increase in shear elastic modulus was observed in the experimental leg compared to the control leg. Since our experiment requires seven time points over 8 h, it is important to consistently maintain the probe pressure and angle throughout the experiments for reliable measurements. Therefore, we have considered two groups and fixed the ultrasound probe using two different setups to investigate echogenicity and SWE considering the echogenicity requires a transverse plane of probe location, while the SWE requires a longitudinal plane of probe location. Since we made comparisons throughout the time points within each group, the subject-specific variation could be considered. In this regard, we could carefully conclude that compared to the echogenicity measure, the shear elastic modulus is more sensitive in detecting CS.

Although this is the first study to investigate echogenicity in leg CS using an in vivo acute atraumatic CS porcine model, echogenicity has been extensively researched to understand muscle morphology and conditions^42–45^. In previous studies, it has been found that muscle atrophy is associated with increased accumulation of fibrous tissues and intramuscular fat, leading to higher ultrasound echogenicity^45^. Previous researchers observed that when ICP increases, echogenicity increases among 50 recreational athletes following the defined exercise program^22^. Based on these results, the pattern of higher echogenicity in the experimental group compared to the control group after reperfusion is a reasonable result; yet, it might not be sensitive enough compared to the shear elastic modulus value for detecting the CS.

In current clinical practice, a standard diagnostic method for CS is to measure the ICP by inserting a thick needle into the muscle^46,47^. Since it is invasive and dangerous, SWE can be an alternative method for safe CS diagnosis. Furthermore, ICP can be monitored continuously by the SWE, enabling physicians to detect CS at an earlier stage. This is crucial to prevent irreversible muscle damage and disability during acute atraumatic CS.

## Material and methods

### Experimental design

Based on our pilot study measuring the shear elastic modulus in pigs, a sample size of seven was determined (power = 0.8; *α* = 0.05; effect size d = 1.6). Therefore, a total of 14 pigs completed the study considering two groups. The 14 pigs were divided into two groups: an echogenicity group (EG) and a shear wave elastography group (SEG), considering that the ultrasound probes should stay at the same position with the same amount of compression throughout the experiments for reducing confounding factors that can be raised owing to changes in the position and contact pressure of the probe on the surface of the pig’s leg skin. The left leg was considered as the experimental leg and the right leg as the control leg for all pigs.

### Animal preparation and anesthesia

The experimental protocol was reviewed and approved by the Institutional Animal Care and Use Committee of Korea University College of Medicine before this study. All experiments were performed under relevant guidelines and regulations. Reporting is guided by the ARRIVE (Animal Research: Reporting of In Vivo Experiments) guidelines. Fourteen conditioned Landrace × Yorkshire × Duroc three-way crossbred pigs were selected (14 males, weight 40 ± 2 kg). Each pig was allowed to rest in a comfortable cage for 3 days before the experiment and allowed easy access to water and food except to food for 8 h before the experiment. A cervical intramuscular injection of azaperone (5 mg/kg), xylazine (2 mg/kg), atropine (0.05 mg/kg), and alfaxalone (4 mg/kg) was used for sedation before anesthesia. An intravenous (IV) catheter was inserted into the dorsal ear vein, and 0.9% NaCl was infused for fluid replacement. Alfaxalone (1.5 mg/kg)/xylazine (0.5 mg/kg) was injected into the dorsal ear vein to induce anesthesia. After induction of anesthesia, the pig was intubated and deep anesthesia was maintained by inhalation of isoflurane gas. Dissection was performed on the paratracheal area to settle a carotid arterial line for central blood pressure monitoring. The carotid arterial line was connected to a pressure-transducing device (AMK 150^®^, Ace Medical Co., Seoul, South Korea). The output of the pressure transducer was displayed on a monitor (BM5, Bionet Co. Berlin, Germany). The vital signs of the pig were continuously monitored. At the end of each experiment, the pig was euthanized with an intravenous injection of potassium chloride (2 mmol/kg).

### Experimental setup

**Figure 7:**
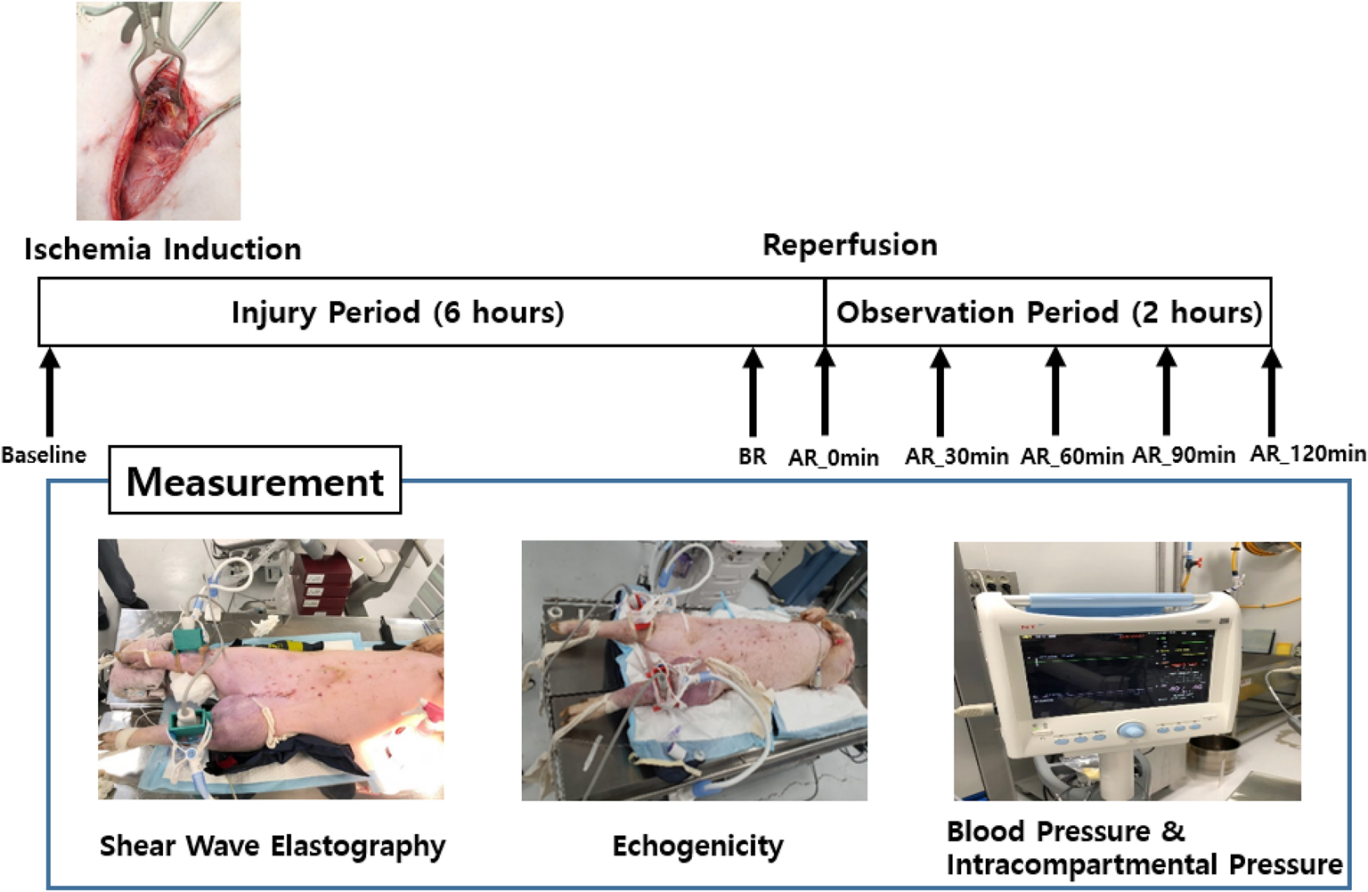
Experimental setup and design.

A beanbag was positioned underneath each pig to maintain the supine position throughout the experiment (Fig. 7). The tibialis anterior muscles of both hind limbs of the pig were selected as the target muscles. Both muscles were identified under ultrasound guidance and marked with a pen. Two ultrasound imaging machines, Aplio i700 (Canon Medical Systems, Tochigi, Japan) were used to quantify the shear modulus of elasticity of the tibialis anterior muscle in the SEG and to quantify the echogenicity and arterial blood flow velocity in the EG. In the SEG, the i18LX5 linear probe was used to collect SWE images of each hind limb at 15MHz. Each probe was placed longitudinally to the marked tibialis anterior muscle belly and angled 90° with the skin surface to maintain the same contact pressure and position throughout the experiment using the three-dimensional (3D) printed probe holder with an adjustable cell phone holder fixed on the experimental table (Fig. 7). In the EG, the PLT-1005BT linear probe was used to collect echogenicity data and arterial blood flow velocity of each hind limb at 11MHz with 82 gain, and each probe was placed transversely to the marked tibialis anterior muscle belly with the other experimental conditions similar to the SEG (Fig. 7). A 24-gauge heparinized IV catheter (Jelco^®^ IV catheter radiopaque, Smith Medical, Ashford, UK) was inserted into the carotid artery to measure the systolic and diastolic central blood pressure. It was connected to a pressure-transducing device (AMK 150^®^, Ace Medical Co., Seoul, South Korea) and continuously monitored each pig’s condition throughout the experiment using a patient monitor (BM5, Bionet Co. Berlin, Germany). A 16-gauge heparinized IV catheter (Jelco^®^ IV catheter radiopaque, Smith Medical, Ashford, UK) was gently inserted in the intermuscular plane between the tibialis anterior and peroneus muscles under ultrasound guidance to measure the ICP of the anterior compartment of the porcine leg, which was also connected to a pressure-transducing device and continuously monitored.

### A surgical procedure to induce the compartment syndrome

Each pig was placed on a surgical table in a supine position. The position was maintained by a beanbag laid beneath the pigs’ back and buttocks, and both hind limbs were restrained by an elastic band in a fully extended position. A 15 cm skin incision was made along the inguinal line in the left hind limb. After subcutaneous dissection, the vascular bundle containing the femoral artery and vein was detected at the intermuscular plane of the medial thigh. Sharp dissection was performed proximally along the femoral artery after incision of the thick inguinal ligament. In the retropelvic space, the common iliac artery (CIA) was observed and tied with a rubber sling to block the blood flow of the CIA. The proximal thigh was tied with an elastic band to block other innominate collateral arteries around the thigh. The tension of the elastic band was adjusted to block all the arterial flow, and the complete blockage of blood flow to the left hind limb was confirmed using Doppler ultrasonography. Previous studies revealed that CS was induced by reperfusion after 6 h of leg ischemia^48,49^. Thereby, we used 6 h for the injury time using leg ischemia. No surgical procedure was performed in the right hind limb since it was considered the control condition.

### Measurement protocol

**Figure 8:**
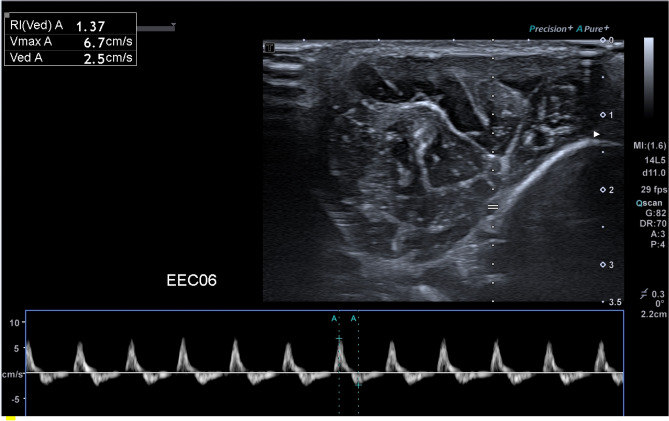
Anterior tibial arterial blood flow velocity.

According to Gifford et al.^48^ and Sanderson et al.^49^, there would not be any changes in muscle during those ischemic periods except possible edema. Thus, a total of seven measurements on both sides were obtained, as indicated in Fig. 7. CS was induced by ligating the CIA for 6 h and reperfusing the CIA. The measurement time points were as follows: (1) right after the ligation of the CIA as baseline; (2) before reperfusion of the CIA indicated as BR; (3) right after reperfusion of the CIA indicated as AR_0 min; (4) approximately 30 min after reperfusion of the CIA indicated as AR_30 min; (5) approximately 60 min after reperfusion of the CIA indicated as AR_60 min; (6) approximately 90 min after reperfusion of the CIA indicated as AR_90 min; (7) approximately 120 min after reperfusion of the CIA indicated as AR_120 min. In the SEG, a minimum of five SWE images were recorded at each time point, along with the systolic and diastolic central blood pressure recordings, and the ICP of both legs. In the EG, one B-mode image and blood flow image of the anterior tibial artery (Fig. 8) were recorded at each point along with the systolic and diastolic central blood pressure recordings, and the ICP of both legs.

### Measurement of shear elastic modulus and echogenicity

**Figure 9:**
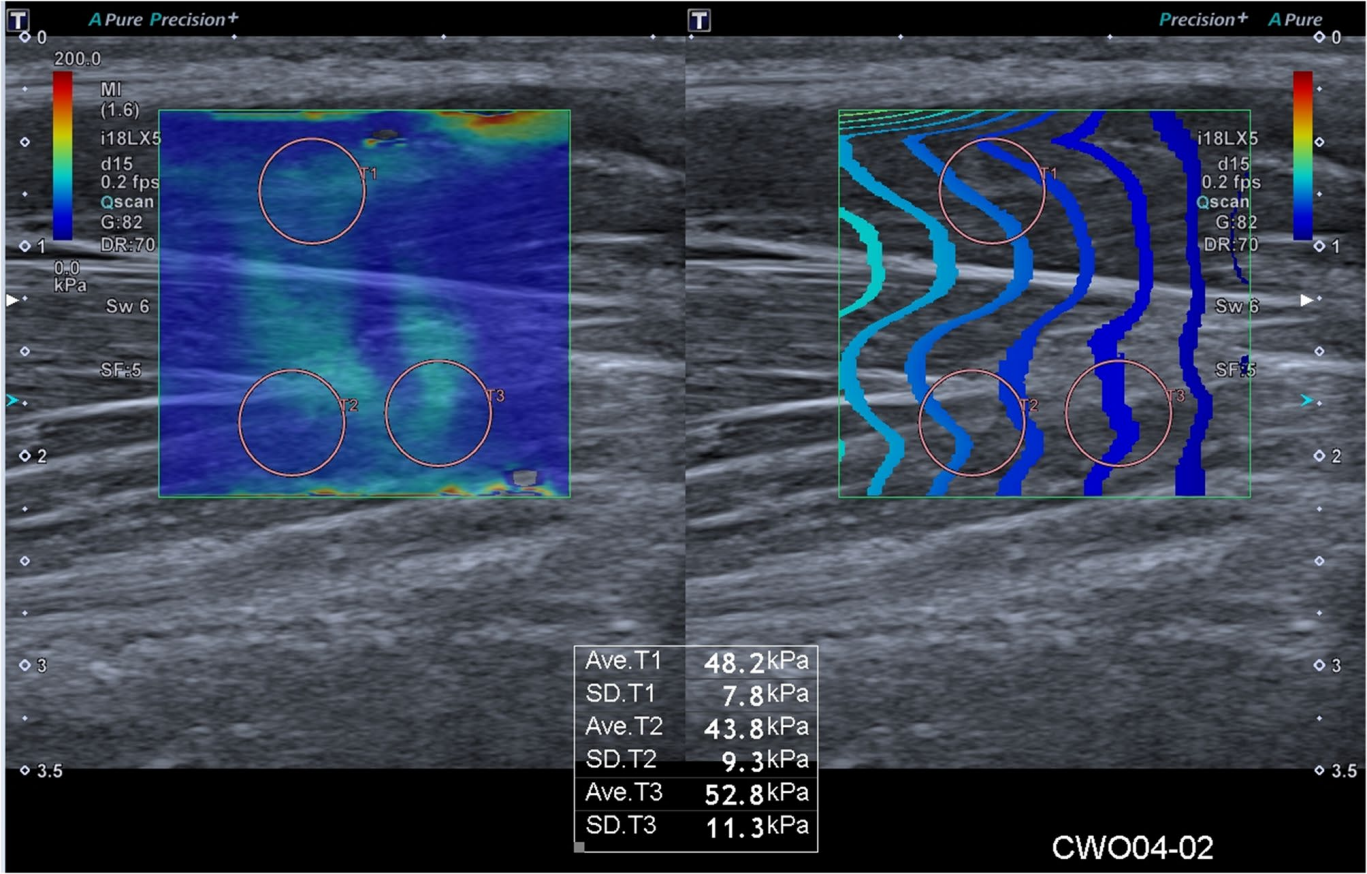
Shear wave elastography image in the region of interest with the calculated value from the ultrasound machine.

The circular regions of interest (ROIs) were set in the color-coded box presentation (2 cm × 2 cm) in the SWE mode. Three random circular ROIs with a diameter of 5 mm were set in the color-coded box (Fig. 9). Random circular ROIs were picked immediately after each ultrasound image was acquired. Then, the shear elastic modulus at the set ROI was automatically calculated using the ultrasound imaging machine. A minimum of 15 circular ROIs was obtained. The mean shear elastic modulus values of the three circular ROIs were calculated for statistical analysis.

**Figure 10:**
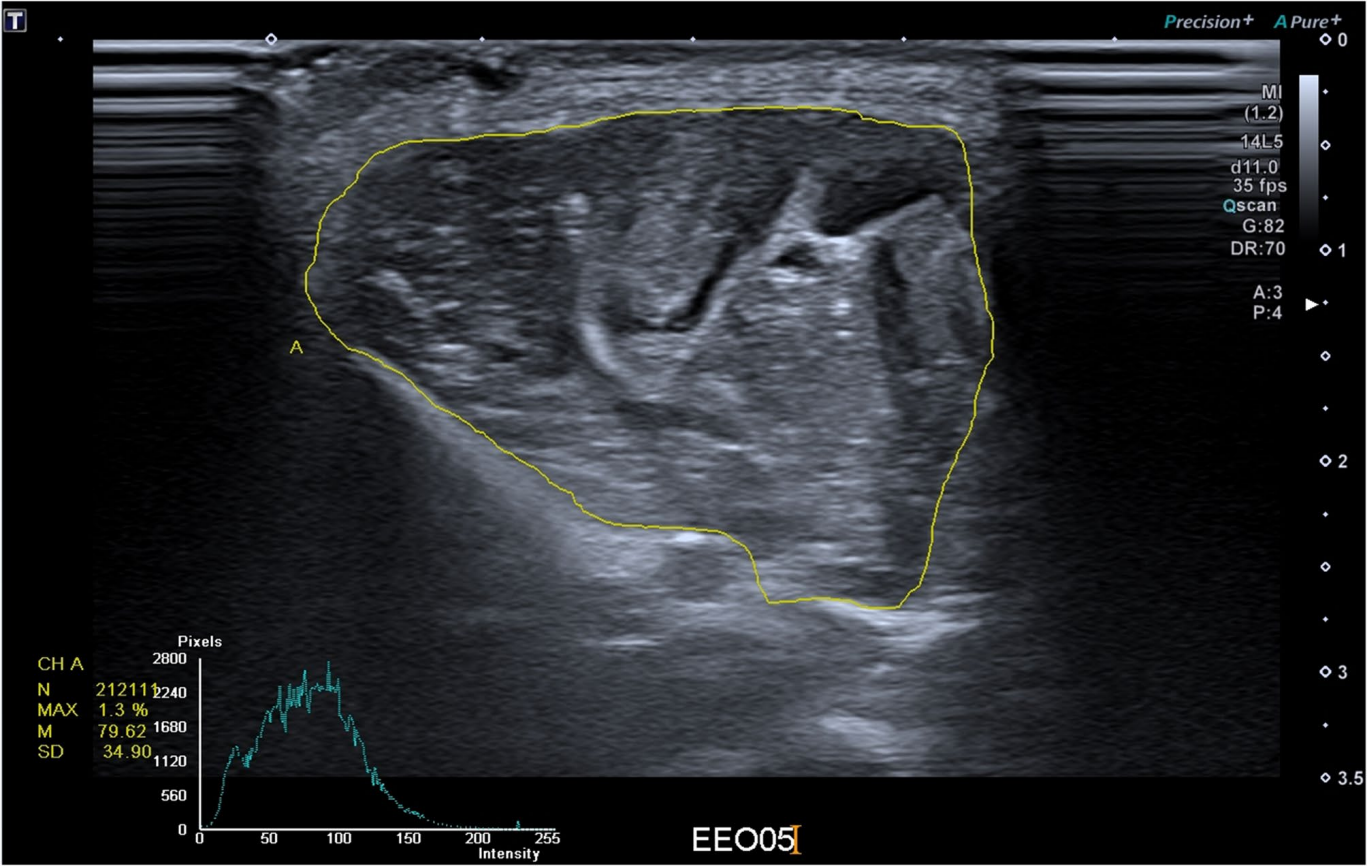
B mode image for calculating echogenicity with the freehand trace of the compartment.

Echogenicity was calculated using the ultrasound imaging machine based on the average value of the brightness in the region of interest (ROI) from the transverse image collected of the yellow trace of the tibialis anterior muscle compartment and the freehand drawing function (Fig. 10).

### Statistical analysis

Due to non-normal data distribution, Wilcoxon rank-sum tests were performed to compare the ICP and perfusion pressure between the experimental legs of the EG and SEG at each time point for the animal model validation. Because our data at each group in each time point were not normally distributed, data were transformed using Aligned Rank Transform (ART) for non-parametric analysis using ARTool and ranked, then two-way repeated ANOVA tests were performed^50^ to determine the effect of condition and time on echogenicity, shear elastic modulus, ICP, and perfusion pressure. The ART is not required for data to be normally distributed and ANOVA can be performed because ART provides accurate non-parametric analysis for both main and interaction effects^50,51^. The main repeated factors were condition (experimental leg vs. control leg) and time point (baseline, BR, AR_0 min, AR_30 min, AR_60 min, AR_90 min, and AR_120 min). The interaction between condition and time was also considered. Perfusion pressure was computed by subtracting the ICP from the diastolic blood pressure. If sphericity was violated, the Greenhouse-Geisser Correction was used for the p-value. When a significant value was found, post hoc analysis with a Bonferroni correction was performed using a paired t-test for the ranked data^50^ investigating the time point, which demonstrated a significant difference between the experimental and control condition by adjusting the p-value by multiplying by 7. In the SEG, Spearman's rank correlation analysis was compared to determine whether there was a correlation between the perfusion pressure and the elastic modulus, between the ICP and the elastic modulus, and between ICP and perfusion pressure using all available data points from all conditions. Similarly, in the EG, Spearman's rank correlation analysis was compared for determining whether there was a correlation between the perfusion pressure and echogenicity, between the ICP and the echogenicity, and between the ICP and perfusion pressure using all available data points from all conditions. Owing to the consistently high ICP throughout the timepoint in one sample, the result was not included in the further analysis. A P-value of less than 0.05 indicated a statistically significant value.
